# Platelet membrane-coated alterbrassicene A nanoparticle inhibits calcification of the aortic valve by suppressing phosphorylation P65 NF-κB

**DOI:** 10.7150/thno.85323

**Published:** 2023-06-26

**Authors:** Bingchuan Geng, Xing Chen, Jiangyang Chi, Fengli Li, Wai Yen Yim, Kan Wang, Chenghao Li, Minghui Xie, Peng Zhu, Zhengfeng Fan, Jiawei Shi, Zhengxi Hu, Yonghui Zhang, Nianguo Dong

**Affiliations:** 1 Department of Cardiovascular Surgery, Union Hospital, Tongji Medical College, Huazhong University of Science and Technology, Wuhan 430022, China.; 2 Hubei Key Laboratory of Natural Medicinal Chemistry and Resource Evaluation, School of Pharmacy, Tongji Medical College, Huazhong University of Science and Technology, Wuhan 430030, China.; 3 Department of Cardiovascular Surgery, Zhongnan Hospital of Wuhan University, Wuhan 430071, China.

**Keywords:** natural product, calcific aortic valve disease, immortalization, NF-κB, nanoparticle

## Abstract

**Rationale:** Calcific aortic valve disease (CAVD) is a leading cause of cardiovascular mortality and morbidity with increasing prevalence and incidence. The pathobiology of CAVD involves valvular fibrocalcification, and osteogenic and fibrogenic activities are elevated in aortic valve interstitial cells (VICs) from diseased valves. It has been demonstrated that activated NF-κB pathway was present in the early stage of CAVD process. There is currently no effective clinical drugs targeting NF-κB pathway for CAVD treatment. Therefore, it is of great clinical significance to seek effective treatments for valve calcification.

**Methods:** In this study, we established immortal human valve interstitial cells (im-hVICs) with pGMLV-SV40T-puro lentivirus. Alizarin red staining and western blotting were performed to evaluate the calcification of immortal VICs supplemented with different compounds. The natural fusicoccane diterpenoid alterbrassicene A (ABA) was found to have potential therapeutic functions. Ribonucleic acid sequencing was used to identify the potential target of ABA. Platelet membrane-coated nanoparticle of ABA (PNP-ABA) was fabricated and the IBIDI pump was used to evaluate the adhesion ability of PNP-ABA. Murine wire-induced aortic valve stenosis model was conducted for in vivo study of PNP-ABA.

**Results:** The natural fusicoccane diterpenoid ABA was found to significantly reduce the calcification of human VICs during osteogenic induction via inhibiting the phosphorylation P65. Runt-related transcription factor 2 (Runx2) and bone morphogenetic protein-2 (BMP2) were down regulated with the treatment of ABA in human VICs. Additionally, molecular docking results revealed that ABA bound to RelA (P65) protein. Phosphorylation of P65 (Ser536) was alleviated by ABA treatment, as well as the nuclear translocation of P65 during osteogenic induction in human VICs. Alizarin red staining showed that ABA inhibited osteogenic differentiation of VICs in a dose-dependent manner. PNP-ABA attenuated aortic valve calcification in murine wire-induced aortic valve stenosis model in vivo.

**Conclusions:** The establishment of im-hVICs provides a convenient cell line for the study of CAVD. Moreover, our current research highlights a novel natural compound, ABA, as a promising candidate to prevent the progression of CAVD.

## Introduction

Calcific aortic valve disease (CAVD) is a progressive disease characterized by lipoprotein deposition, chronic inflammation, and thickening of the valve leaflets 1. CAVD remains one of the most prevalent and costly heart disorders in developed countries, affecting 4.6% of people over 75 years and 6% of adults over 65 years 2. Currently, there is no effective medical treatment for CAVD other than surgery or interventional valve replacement. Numerous studies have shown that CAVD is a regulated pathological process mediated by phenotypic changes in valve interstitial cells (VICs), the most abundant cell type in valve tissue. Chronic stress is thought to cause prolonged activation of VICs 3. Statins and angiotensin-converting enzyme inhibitors have long been highly promising, but many of their large clinical trials have confirmed that neither treatment is effective in slowing disease progression 4, 5. Therefore, it is of great clinical significance to seek effective treatments for valve calcification, such as using systemic drugs to modulate the early inflammatory response, which may effectively prevent the occurrence of heart valve calcification.

Primary human VICs (p-hVICs) have a limited proliferative capacity and heterogeneous phenotype. They comprise at least four cell types, including fibroblasts, myofibroblasts, cells resembling smooth muscle cells, and stem cells 6. Additionally, p-hVICs have several limitations, including heterogeneity, limited proliferative capacity, and individual differences 7. The development of immortal cell lines would extend the life span of these cells and provide an almost unlimited supply of cells for complex, continuous, long-term studies, and inter-laboratory comparisons of findings 8. This study established immortal human VICs (im-hVICs) to screen for potential anticalcific compounds from our in-house natural products library. Fusicoccanes represent an insufficiently explored family of terpenoids characterized by a 5-8-5-fused carbocyclic (dicyclopenta[*a*,*d*]cyclooctane) skeleton, and are reported to show remarkable phytohormone-like activities resulting from interactions with the plant 14-3-3 proteins relevant for drug discovery 9. In this work, alterbrassicene A (ABA, compound **6**), a rearranged fusicoccane diterpenoid defined by a novel 5/9/4-fused carbocyclic skeleton isolated from fungus *Alternaria brassicicola* with significant anti-inflammatory activity 10, was identified as a potential therapeutic compound to attenuate the calcification of human VICs. Osteogenic induction medium (OM) containing organic β-glycerophosphate, dexamethasone, and ascorbic acid is a classic calcification and inflammatory induction medium in CAVD. Changes in global gene expression in im-hVICs induced by OM with or without ABA were further analyzed by a high-throughput ribonucleic acid (RNA) sequencing. The tumor necrosis factor-alpha (TNF-α)-mediated nuclear factor kappa-light-chain-enhancer of activated B cells (NF-κB) pathway has been demonstrated to be associated with the anticalcific effect of ABA. Molecular docking based on computer simulations suggests that the RelA (P65) protein is a potential target for ABA.

Cell membrane-coated nanoparticle delivery systems have emerged as promising therapeutic platforms 11. Platelet membrane-coated nanoparticles (PNPs) stand out for their unique ability to target both passively and actively toward effective drug targeting. PNPs exhibit a circulation half-life of approximately 30 h in vivo and express a set of unique surface receptors that dynamically adhere to damaged vasculature, tumor cells, and pathogenic bacteria 12-14. In vivo study showed that PNPs could effectively adhere to sclerotic aortic valves under pathological shear stress and co-localized with von Willebrand factor (vWF), collagen and fibrin. Accordingly, PNPs are attracting increasing attention and have shown great promise for clinical applications for the treatment of atherosclerosis and sclerotic aortic valve disease 15. This study attempted to fabricate the alterbrassicene A-loaded platelet membrane-coated nanoparticle (PNP-ABA) to facilitate drug delivery in the aortic valve regions. The in vitro cellular uptake and in vivo anticalcific activity of PNP-ABA were evaluated. Our current study highlights ABA as a promising candidate to prevent the progression of CAVD.

## Materials and Methods

### Immortal human VICs establishment

Healthy human aortic valve leaflets were collected from the explanted hearts of patients who underwent heart transplant procedures for dilated cardiomyopathy. Primary hVICs were isolated from human aortic valves, as previously described 16. The separated p-hVICs were cultured in high glucose Dulbecco's modified Eagle's medium (DMEM, Gibco, Invitrogen, Carlsbad, CA, USA) supplemented with 10% fetal bovine serum (FBS) (Gibco), 100 U/mL penicillin, and 100 µg/mL streptomycin (all Sigma-Aldrich, Buchs, Switzerland) at 37 °C, 95% air, and 5% CO_2_ in a humidified incubator. Primary hVICs (passage 2) were cultured to 50-60% confluence and infected with pGMLV-SV40T-puro lentivirus (NewgainBio, Wuxi, China) at a multiplicity of infection of 80 supplemented with 5 µg/mL polybrene (Sigma-Aldrich, Buchs, Switzerland). Infected hVICs were cultured for 4 days, and the medium was changed every 2 days, followed by culturing with medium supplemented with 1 µg/mL puromycin for 4 days. Im-hVICs were transferred to a normal medium and cultured until passage 10. Primary hVICs used in the experiments were passages 2-4, and im-hVICs were passaged 10-20.

### Human VICs calcification induction

An OM containing 2% FBS, 10 mM β-glycerophosphate, 100 nM dexamethasone, and 50 mg/mL ascorbic acid (Sigma-Aldrich) was used to induce calcification. Cells were starved overnight in DMEM containing only 2% FBS and treated with either OM only or OM supplemented with the indicated compounds. For western blotting and immunofluorescence staining, cells were treated for 5 days before analysis. For alizarin red staining, the cells were cultured for 28 days with the indicated treatment before staining.

### Western blotting analysis

Total proteins were extracted from cells using radioimmunoprecipitation assay buffer containing protease and phosphatase inhibitor cocktails (New Cell & Molecular Biotech Co., Suzhou, China). The denatured proteins were run on 4-20% pre-made sodium dodecyl-sulfate/polyacrylamide gels, and the separated proteins were transferred to polyvinylidene fluoride membranes (Merck Millipore, Berlin, Germany). Membranes were blocked with 5% nonfat dry milk in Tris-buffered saline with 0.05% Tween-20 (TBST), followed by probing with the primary antibody and incubation at 4 °C overnight. The membranes were then washed with TBST and probed with a secondary antibody. Protein expression was detected using a Bio-Rad imaging system with a chemiluminescent substrate (Thermo #34080). The following antibodies were used: runt-related transcription factor 2 (Runx2) (CST #8486), bone morphogenetic protein 2 (BMP2) (Abmart #TA5163), glyceraldehyde 3-phosphate dehydrogenase (CST #5174S), osteocalcin (OCN, Millipore #AB10911), P65 (Abmart #TU329612), pP65-Ser536 (Abmart #TA2006), and P52 (Proteintech #15503-1-AP).

### Immunofluorescent staining

The cells were washed with PBS and fixed with 4% paraformaldehyde for 15 min at room temperature. The fixed cells were permeabilized with 0.3% Triton X-100 for 10 min at room temperature, followed by washing twice with PBS. Cells were blocked with 5% donkey or goat serum (the species where the secondary antibodies were raised) for 1 h and then incubated with primary antibodies diluted in blocking buffer containing phosphate-buffered saline with 0.05% Tween-20 (PBST) at 4 °C overnight. Cells were thoroughly washed with PBST three times and then incubated with secondary antibodies conjugated with fluorescent dyes for 1 h at room temperature, followed by washing with PBST three times. The stained cells were then treated with mounting media containing DAPI (Fluoromount-G, SouthernBiotech, #SBA-0100-20) and sealed with coverslips. Mouse aortic valve sections were obtained from cryoblocks. Staining slides were visualized using either a panoramic MIDI scanner or an Olympus FV3000 confocal system. The following antibodies were used: vimentin (Abcam, ab8978, 1:200 dilution), Runx2 (Santa Cruz, sc-101145, 1:200 dilution), pP65-Ser536 (Abmart, TA2006, 1:100 dilution), BrdU (Abmart, T62051, 1:1000 dilution), anti-rabbit IgG Alexa Fluor647 (Invitrogen, A21244, 1:1000 dilution), anti-mouse IgG Alexa Fluor488 (Invitrogen, A11029, 1:1000 dilution), and anti-mouse IgG Alexa Fluor647 (Invitrogen, A31571, 1:1000 dilution).

### Crystal violet staining

The p-hVICs and im-hVICs were plated in 6-well dishes (500 cells per dish) and cultured for 10 days. Afterward, the cells were washed with PBS and fixed with 4% paraformaldehyde for 10 min at room temperature. Subsequently, the fixed cells were incubated with crystal violet staining solution (Servicebio, G1014) for 10 min at room temperature. Finally, the stained cells were washed with running water and visualized under a Zeiss microscope.

### Alizarin red staining

Alizarin red staining was performed to detect calcium deposits. Briefly, VICs were washed twice with PBS and fixed for 10 min in 4% paraformaldehyde. After incubation with alizarin red solution (Servicebio, G1038) for 30 min at 37 °C, the excess dye was removed by washing under running water. Subsequently, stained cells were mounted using a xylene-based mounting medium and imaged. The calcified area was calculated using ImageJ (National Institutes of Health) and averaged for independent biological replicates.

### Von Kossa staining

Cryo-sections from the mouse aortic valve were rinsed with distilled water. The slices were immersed in 1% silver nitrate for 30 min under intense sunlight and then washed three times with deionized water. Subsequently, 5% sodium thiosulfate (Sigma-Aldrich) was added for 5 min to remove unreacted reagents. Afterward, the calcium phosphate salts were visualized using black staining.

### Cell proliferation assay

Cell viability was evaluated using the Cell Counting Kit-8 (CCK-8) kit (Absin, abs50003). The primary and immortal hVICs were seeded into 96-well plates and cultured overnight to allow attachment. After serum starvation for 24 h, FBS was added to the medium. At 0, 24, 48, 72, and 96 h, cells were incubated with CCK-8 working solution for 2 h at 37 °C. After that, the optical density at 450 nm was analyzed using a microplate reader (Thermo Fisher Scientific).

### Apoptosis assay

To detect apoptosis, P2 of p-hVICs and P20 of im-hVICs were harvested from culture dish. The cells were labeled using Annexin V-FITC/Propidium Iodide Apoptosis Detection Kit for 15 min under no light condition. The stained cells were then quantified by a flow cytometer.

### Wound scratch assay

Briefly, cells were plated in a 6-well plate and cultured overnight to allow attachment. After serum starvation for 12 h, the cells were scratched with a pipette tip (20 μL) and cultured in a complete medium. Subsequently, wounds were observed using a Zeiss microscope at 0-72 h along the scratch.

### Cell viability assay

The half maximal inhibitory concentration (IC_50_) value of ABA on im-hVICs was determined using the CCK-8 kit, according to the manufacturer's instructions. Cells were seeded into 96-well plates at a density of 10000 cells per well and then treated with ABA for 72 h at different concentrations. Next, the cells were incubated in fresh medium (100 μL), and CCK-8 solution (10 μL) for 2 h at 37 °C, and optical density was measured at 450 nm using a microplate reader (Thermo Fisher Scientific).

### Detection of mRNA profiles

RNA-sequencing (RNA-seq) technology was used to investigate changes in cell mRNA profiles among the p-hVICs, im-hVICs, and different treatments. Isolated RNA was sent to BGI Co., Ltd. (Shenzhen, Guangdong, China) for RNA-seq performed on a BGISEQ-500, and all samples were replicated three times for confirmation purposes. Sequencing results were further analyzed using the "R Project" in order to identify differentially expressed genes and perform gene ontology and Kyoto Encyclopedia of Genes and Genomes (KEGG) pathway enrichment analysis.

### Molecular docking analysis

The molecular docking technology completed by AutoDock Vina 1.1.2 software 17 was applied to predict the binding modes between small molecules and proteins. Before starting molecular docking, the protein crystal structure was obtained from the protein data bank (PDB) and universal protein resource databases. PDB and UniProt IDs are as follows: RelA (1NFI), NF-κB1 (3GUT), NF-κB2 (1A3Q), RelB (Q01201), and c-Rel (Q04864). The 3D structure of the target small molecule was constructed using Chem3D, and the small molecule was energy-minimized using the Merck Molecular Force Field. Before formal docking began, PyMol 2.5 software 18 was used for protein preparation, including dehydrogenation, removal of water, and non-ligand small molecules. The docking box was defined to enclose the protein activity pocket. ADFRsuite 1.0 19 was used to convert small molecules and receptor proteins in PDB format to Protein Data Bank, Partial Charge (Q), & Atom Type (T) format. Finally, molecular docking analysis was performed. During docking, the conformational search detail was set to 32, and the other parameters were set to default settings. The conformation with the highest affinity score was output as the correct conformation and visualized using PyMol 2.5.

### Preparation of PNP-ABA

Platelets were collected from rat platelet-rich plasma as described previously 20. The platelet membrane was derived by a repeated freeze-thaw process, followed by centrifugation at 4000 g for 3 min. The supernatants were removed, and platelet membrane pellets were resuspended in ddH_2_O. Platelet membrane suspensions were then sonicated 30 times at a power of 100 W at 40 kHz. ABA and platelet membrane suspensions were freeze-dried overnight and resuspended in ddH_2_O. ABA and platelet membranes were combined by gentle stirring, and the mixture was incubated at room temperature for 15 min. The properties of the platelet membrane-coated ABA nanoparticles were determined by scanning electron microscopy (SEM) and mass spectrometry (LS-MS/MS).

### Isolation and culturing of primary mouse VICs

Primary mouse valve interstitial cells (mVICs) were isolated from mouse aortic valves as previously described 21. Briefly, aortic valves were collected from an 8-week-old adult male C57BL/6-J mouse. Isolated valves were washed with 5 mL of cold HEPES (10 mM) supplemented with antibiotics (1% penicillin-streptomycin) to remove blood. The valves were incubated with 1 mg/mL collagenase type I for 30 min at 37 °C with continuous shaking and then transferred to 4.5 mg/mL collagenase type I. Samples were incubated at 37 °C under continuous agitation for 35 min and centrifuged at 150 g for 5 min at 4 °C. Resuspend the pellet in 1 mL of complete medium and plate the cells. When the cells were 70% confluent, the trypsinized cells were plated in dishes for subsequent experiments.

### Adhesion assay using the IBIDI pump

The IBIDI pump system, manufactured by IBIDI (Cat. No. 10902, Germany), is a suitable tool for cultivating cells under flow conditions in order to replicate the biomechanical environment. To this end, mVICs were cultured on µ-slide I 0.4 Luer IBIDI treat chambers (IBIDI, Cat No. 80176, Germany) until they reached confluency, and were subsequently subjected to low unidirectional stress (5 dyn/cm^2^) using the IBIDI pump system for a duration of 24 h. During dynamic culturing, the cells were maintained in a medium containing either ABA or PNP-ABA. Following the culture period, the cells were harvested and submitted for mass spectrometry analysis.

### Animal studies

Mice were purchased from Shulaibao (Wuhan) Biotechnology Co., Ltd. ABA and PNP-ABA were intravenously injected into the adult male C57BL/6-J mice, and aortic roots were collected at different time points after injection. The ABA concentration in the mouse aortic root was determined using mass spectrometry (LS-MS/MS). As previously described, a wire-induced aortic valve stenosis model was used in the study 22. Briefly, the adult males C57BL/6-J (eight weeks old, weighing 20-25 g) were randomly divided into two groups. Animals were maintained on a 12/12 h light/dark cycle and received water and a standard rodent diet. Mice were anesthetized by intraperitoneal injection of 100 mg/kg ketamine and 10 mg/kg xylazine by body weight. The right carotid artery was exposed by blunt dissection, and blood flow was stopped using ligature loops. A small incision was made in the right carotid artery, and a guide wire (0.36 mm diameter) was inserted through the small incision. The wire position was confirmed by echocardiography, and the wire was carefully inserted into the left ventricle. Scratch the aortic valve with the body of the wire 50 times. After the injury, the wire was removed, and the carotid artery was ligated. The sham group underwent the same procedure, but the wire was inserted into the right carotid artery and not advanced across the aortic valve into the left ventricle. The experimental group was intravenously injected with PNP-ABA (50 mg/kg) every four days post-surgery. The aortic valve peak velocity was assessed in vivo using transthoracic echocardiography with an 18-38 MHz phased-array probe (MS400) connected to a Vevo 2100 imaging system. The entire heart was collected for further analysis.

### Statistical analysis

A student's t-test was used to determine the statistical difference between groups.

## Results

### Immortal human VICs function similarly to primary human VICs

**Figure 1:**
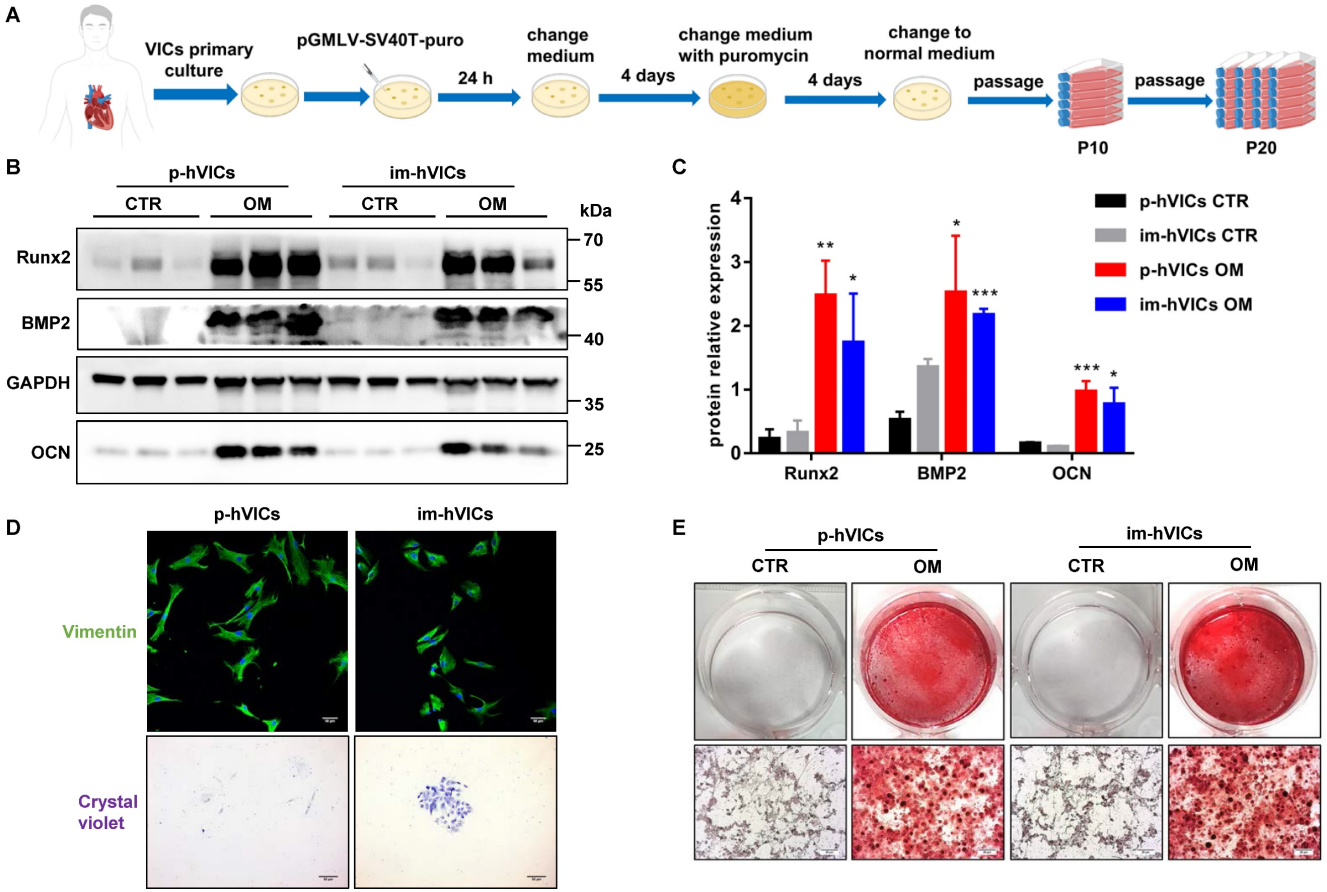
** Immortal human VICs function similarly to primary human VICs.** (**A**) The flow chart for establishment of immortal human VICs (im-hVICs); (**B**, **C**) Western blotting (WB) analysis and WB quantification of Runx2, BMP2 and OCN protein expression at day 5 of osteogenic induction in both im-hVICs and p-hVICs (n = 6 per group. Data are presented as the mean ± SEM, *P<0.05, **P < 0.01, ***P < 0.001); (**D**) Immunofluorescent staining was used to examine p-hVICs marker vimentin in both im-hVICs and p-hVICs, and representative crystal violet staining of clone formation is shown (scale bar: 50 µm); (**E**) Im-hVICs and p-hVICs were stained with alizarin red after osteogenic induction (scale bar: 20 µm).

We first established the im-hVICs line using the pGMLV-SV40T-puro lentivirus (**Figure 1A**). Im-hVICs still expressed vimentin (**Figure 1D**), which is a hallmark of p-hVICs. Im-hVICs and p-hVICs were cultured in an osteogenic induction medium for five days and analyzed by western blotting. Runx2, BMP2, and OCN were significantly upregulated in both cell lines (**Figure 1B-C**), as well as calcium deposits, as shown by alizarin red staining (**Figure 1E**). Single im-hVIC could grow into a cell clone after 10 days of culturing, whereas p-hVIC could not (**Figure 1D**). No significant difference was found between im-hVICs and p-hVICs in a scratch wound healing assay (**Figure S1A-B**). The proliferation of two cell lines was assessed using the CCK-8 assay. As expected, im-hVICs grew faster than p-hVICs did (**Figure S1C**). For cultured P2 p-hVICs, 27.32% cells were BrdU positive. However, 35.48% P20 im-hVICs were BrdU positive (**Figure S1D-E**). Apoptotic cells in two groups are lower than 1% (**Figure S1F-G**). RNA sequencing results indicated that growth and development signals were enhanced in im-hVICs compared to p-hVICs. Furthermore, ossification pathways were activated in both cells after culturing in OM (**Figure S2A-B**).

### ABA was identified as a potential anticalcific compound

**Figure 2:**
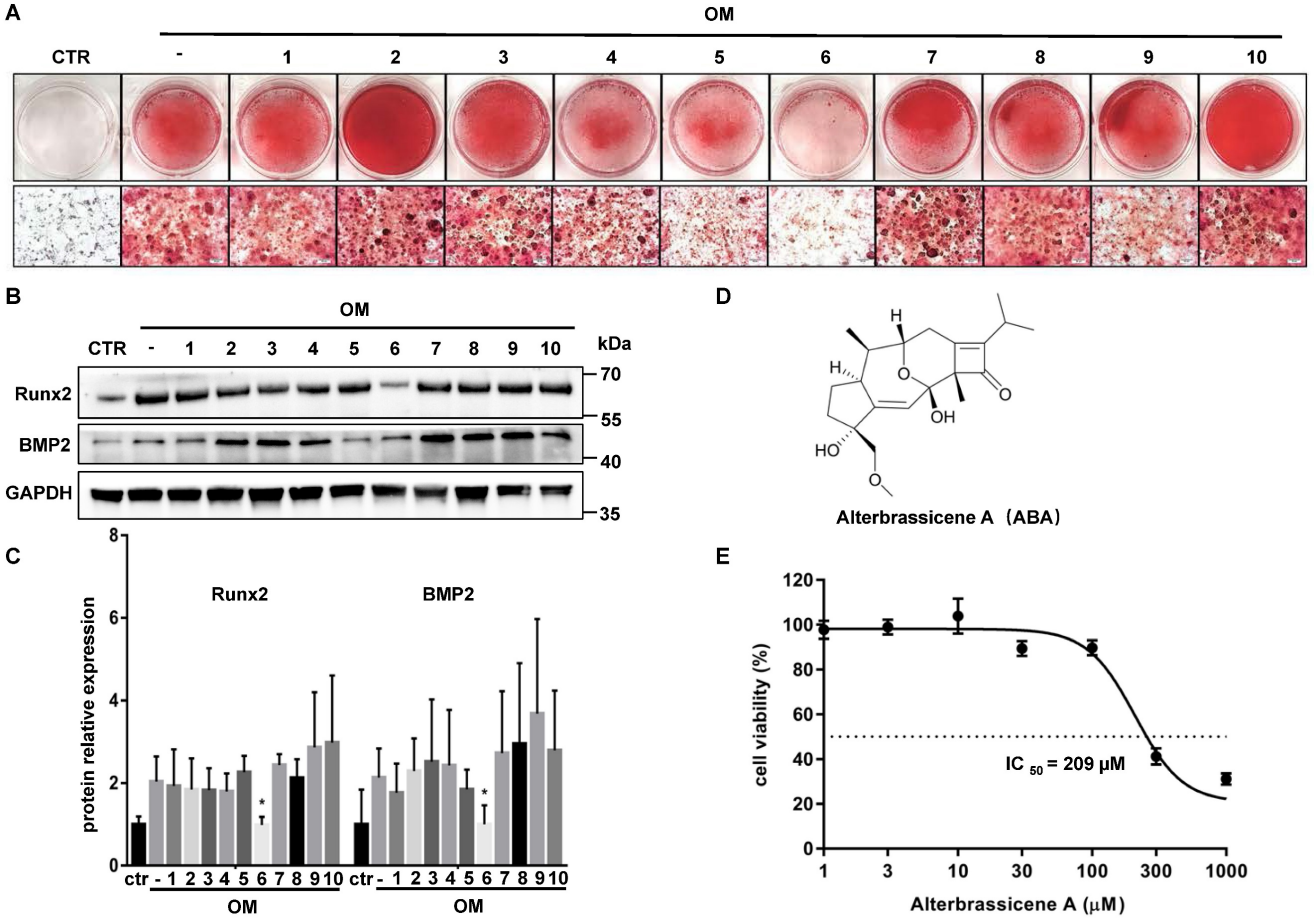
** ABA is identified as a potential anticalcific compound.** (**A**) Ten different compounds (10 µM) were screened by evaluating the effect of anti-calcification treatment with stained alizarin red (scale bar: 20 µm); (**B**, **C**) WB analysis and WB quantification of Runx2 and BMP2 protein levels after ten different compounds (10 µM) used in OM-cultured VICs (n = 5 per group. Data are presented as the mean ± SEM, *P<0.05); (**D**) The chemical structure of compound 6; (**E**) The effects of the indicated compounds on cell viability were examined in im-hVICs.

We performed drug screening using im-hVICs because of the high consistency of the immortal cell line. Im-hVICs were cultured in an osteogenic induction medium supplemented with 10 different compounds. Alizarin red staining showed that compound **6** (Alterbrassicene A, ABA) could reduce calcium deposits during osteogenic induction (**Figure 2A**). Runx2 and BMP2 were also significantly downregulated by ABA treatment during the osteogenic induction of im-hVICs (**Figure 2B-C**). The IC_50_ value of ABA in im-hVICs was 209 µM in the CCK-8 cell viability assay (**Figure 2D-E**).

### ABA-induced anticalcific response in im-hVICs involved NF-κB pathway activation

**Figure 3:**
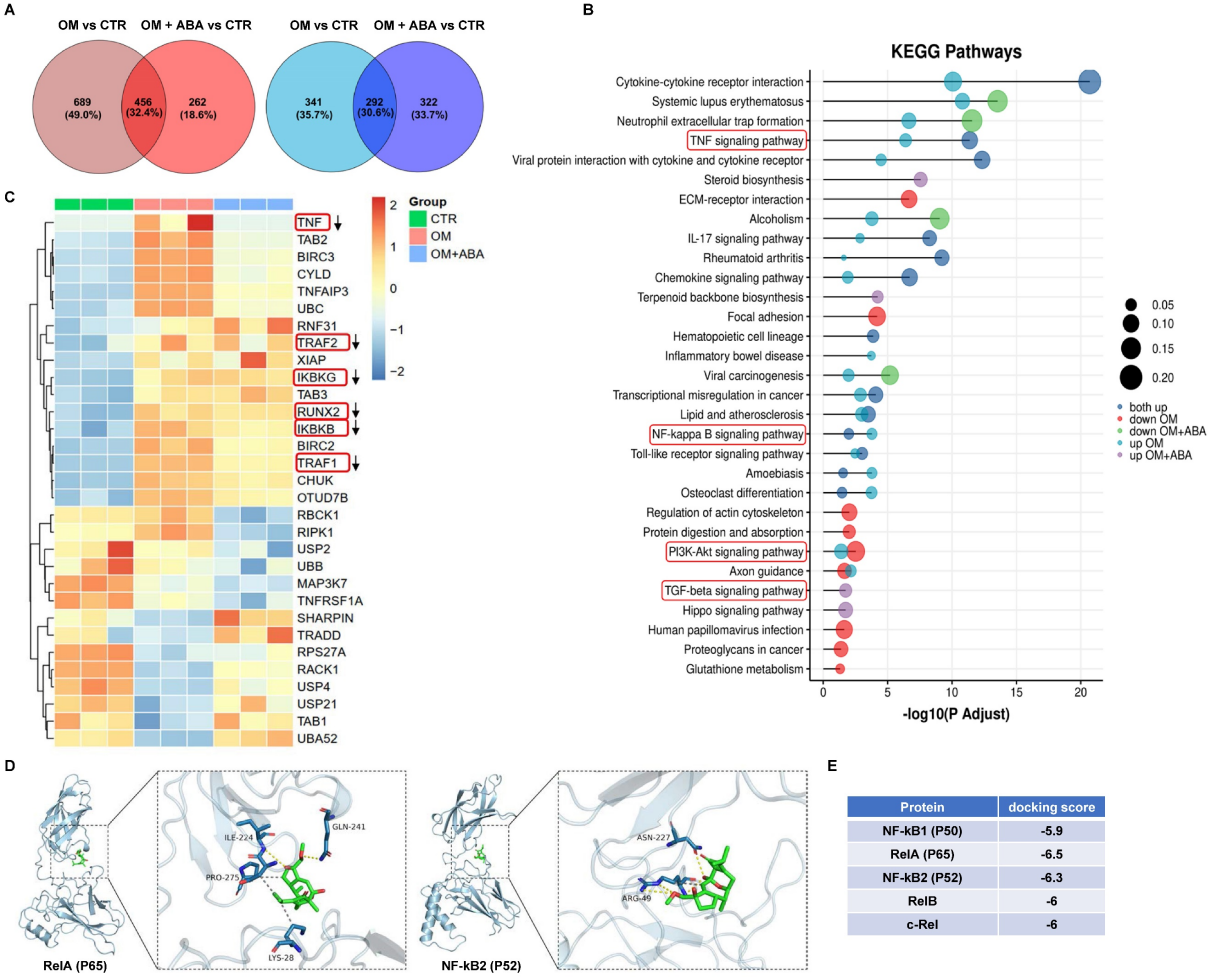
** ABA induced anticalcific response in im-hVICs involved NF-κB pathway activation.** (**A**) The Venn diagram displays the number of regulated genes shared between ABA compounds and OM groups in upregulation (Red) and downregulation (Blue); (**B**) KEGG pathway analysis of genes regulated by ABA compounds; (**C**) Heatmap representing the selected differentially expressed genes (DEGs) across all three groups of samples; (**D**) ABA docked with P65 and P52; (**E**) Results of molecular docking score.

RNA-sequencing analysis was used to investigate changes in the mRNA profiles of cells cultured under osteogenic induction with or without ABA. KEGG pathway analysis indicated that the TNF, NF-κB, phosphatidylinositol 3-kinase-serine/threonine kinase (PI3K-AKT), and transforming growth factor beta (TGF-β) signaling pathways were enriched (**Figure 3A-B**). Further analysis showed downregulation of TNF, TRAF2, TRAF1, Runx2, IKBKG, and IKBKB in response to ABA treatment during osteogenic induction (**Figure 3C**). Molecular docking analysis indicated that ABA better bound to RelA (P65) and NF-κB2 (P52) (**Figure 3E**). The amino acid-binding sites of P65 and ABA were isoleucine-224, glutamine-241, proline-275, and lysine-28 (**Figure 3D**). The binding sites of P52 and ABA were asparagine-227 and arginine-49, respectively (**Figure 3D**).

### ABA reduced the phosphorylation of RelA

**Figure 4:**
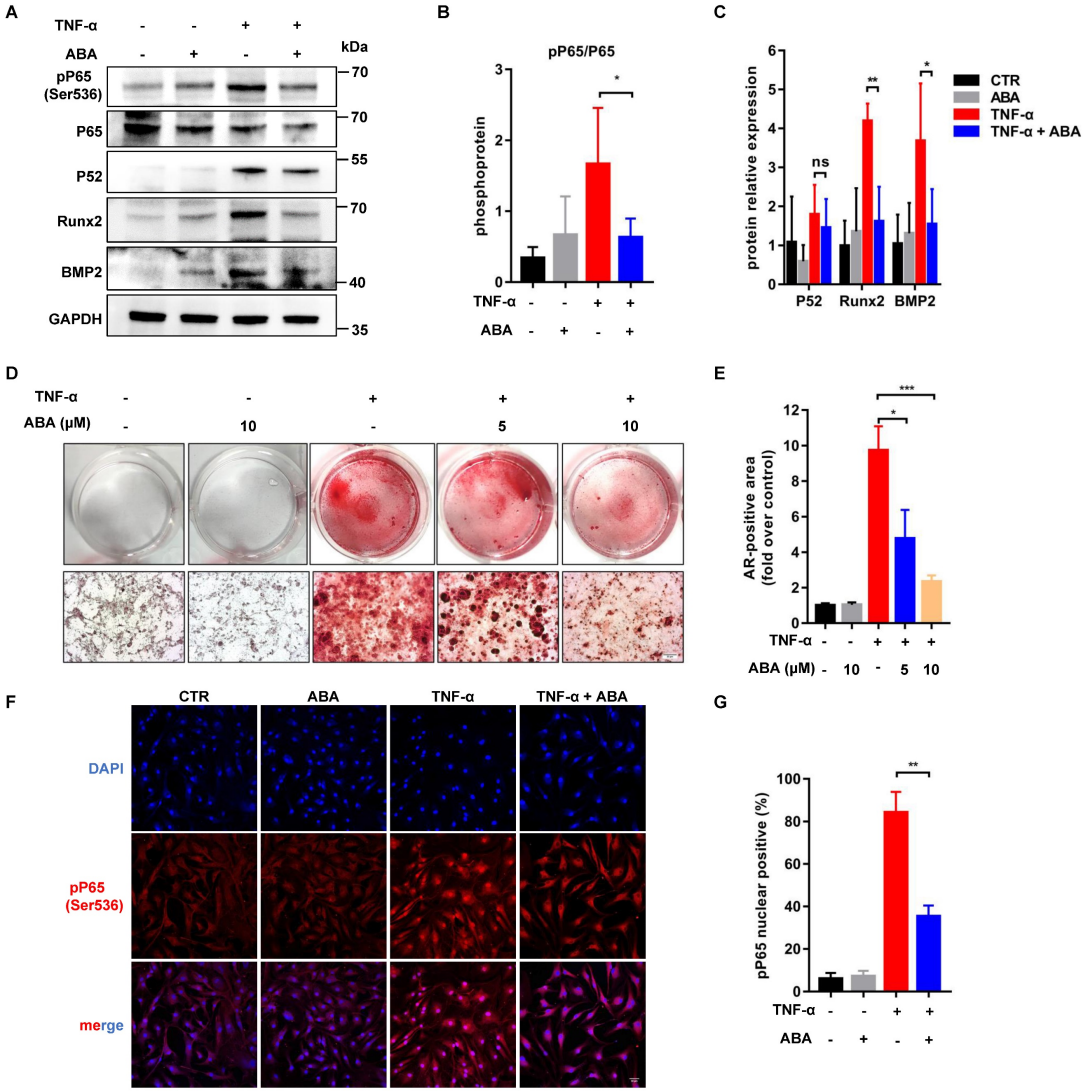
** ABA reduces the phosphorylation of RelA.** (**A**, **B**, **C**) WB analysis and quantification of Runx2, BMP2, P65, P52 and phosphorylation of P65 proteins levels after ABA compounds used in TNF-α cultured im-hVICs (n = 4 per group. Data are presented as the mean ± SEM, ns indicates no significant, *P<0.05, **P < 0.01); (**D**) The dose-dependent treatment effect of ABA compound was detected by Alizarin Red (scale bar: 20 µm); (**E**) The alizarin red positive area were quantified in im-hVICs (n = 6 per group. Data are presented as the mean ± SEM, *P<0.05, ***P < 0.001); (**F**) Immunofluorescence analysis of p65 nuclear translocation (scale bar: 50 µm); (**G**) The quantification of immunofluorescence analysis. (n = 6 per group. Data are presented as the mean ± SEM, **P < 0.01)

Im-hVICs were treated with TNF-α (20 ng/mL) for osteogenic induction. Western blotting results showed that Runx2, BMP2, P52, and phosphorylation of P65 were increased after treatment with TNF-α. ABA (10 µM) significantly downregulated Runx2, BMP2, and phosphorylation of P65 but not P52 expression (**Figure 4A-C**). Compared to the control group, treatment with 5 or 10 μM ABA also effectively reduced calcium deposits (**Figure 4D-E**). Immunofluorescent staining showed that the nuclear translocation of phosphorylation P65 was significantly reduced by treatment with 10 µM ABA (**Figure 4F-G**). Three weeks treatment of TNF-α could fully induce the calcification of im-hVICs (**Figure S3B-C**). However, calcium deposits of the calcified im-hVICs could not be reduced with the treatment of 10 µM ABA as shown by alizarin red staining (**Figure S3A-C**).

### PNP-ABA could better target mVICs

**Figure 5:**
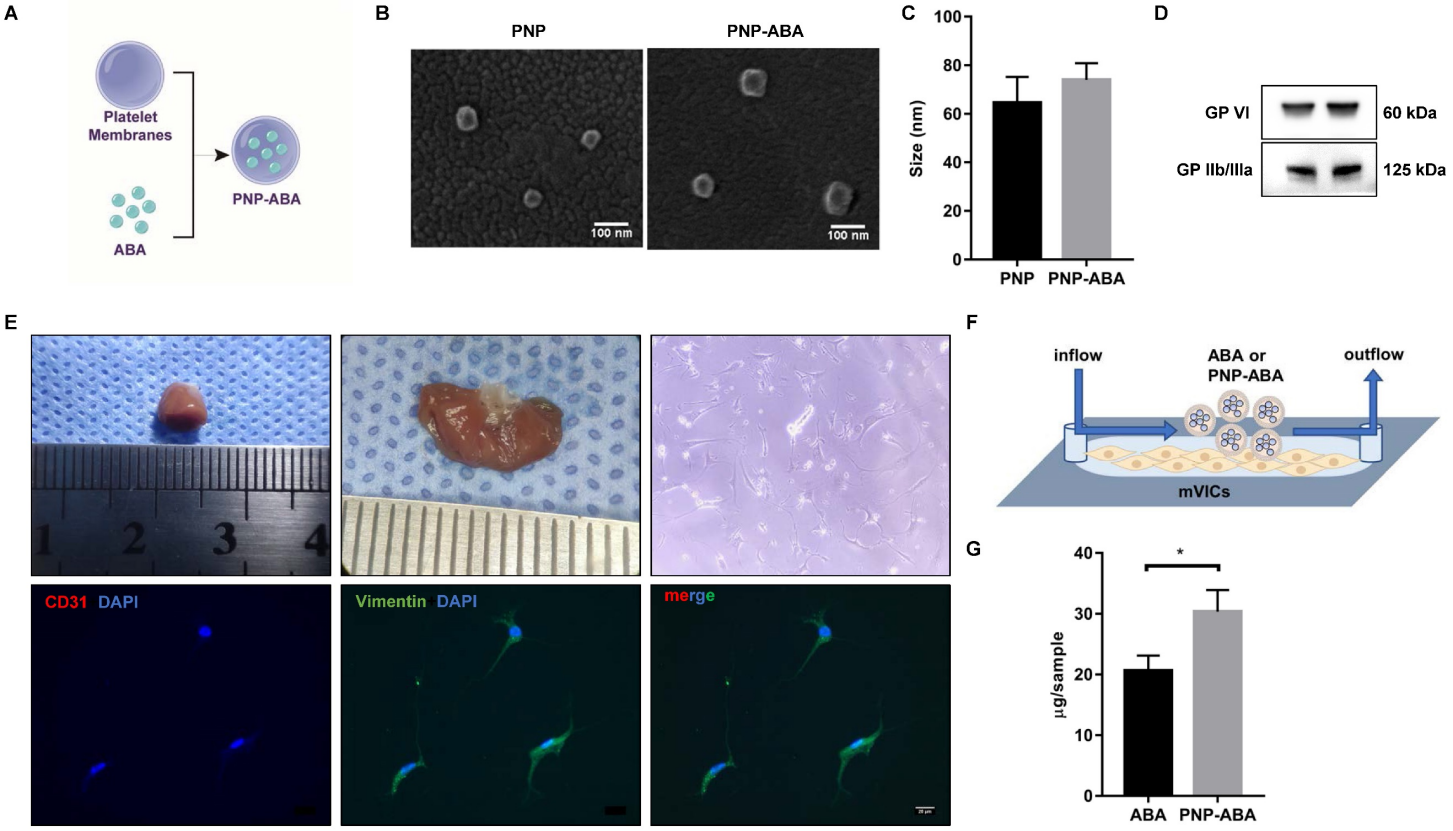
** Platelet membrane-coated ABA can better target mVICs.** (**A**) Fabrication process of the PNP-ABA; (**B**, **C**) Scanning electron microscope images and size distribution of PNP and PNP-ABA samples (scale bar: 100 nm); (**D**) WB analysis of PNP and PNP-ABA for characteristic platelet membrane markers; (**E**) Photograph of the mice heart tissue and valve sample is from left to right. The isolated mVICs were stained with CD31, Vimentin and DAPI (scale bar: 20 µm); (**F**) The IBIDI instrument was used to compare the extent of binding between ABA and PNP-ABA in mVICs; (**G**) The remaining compounds were detected and quantified. (n = 6 per group. Data are presented as the mean ± SEM, *P<0.05)

Platelet membrane-coated ABA (PNP-ABA) was fabricated according to a previous description 20 (**Figure 5A**). Scanning results showed that the sizes of bare platelet membrane nanoparticles (PNP) and PNP-ABA were similar (**Figure 5B-C**). Western blotting result indicated that PNP and PNP-ABA samples expressed platelet membrane markers of GP VI and GP IIb/IIIa (**Figure 5D**). Primary mouse VICs were isolated, and the immunofluorescence staining showed no valve endothelial cell contamination (**Figure 5E**). The IBIDI experiment showed that PNP-ABA was a better combination with mVICs than ABA (**Figure 5F-G**).

### ABA attenuated calcification in murine wire-induced aortic valve stenosis model

**Figure 6:**
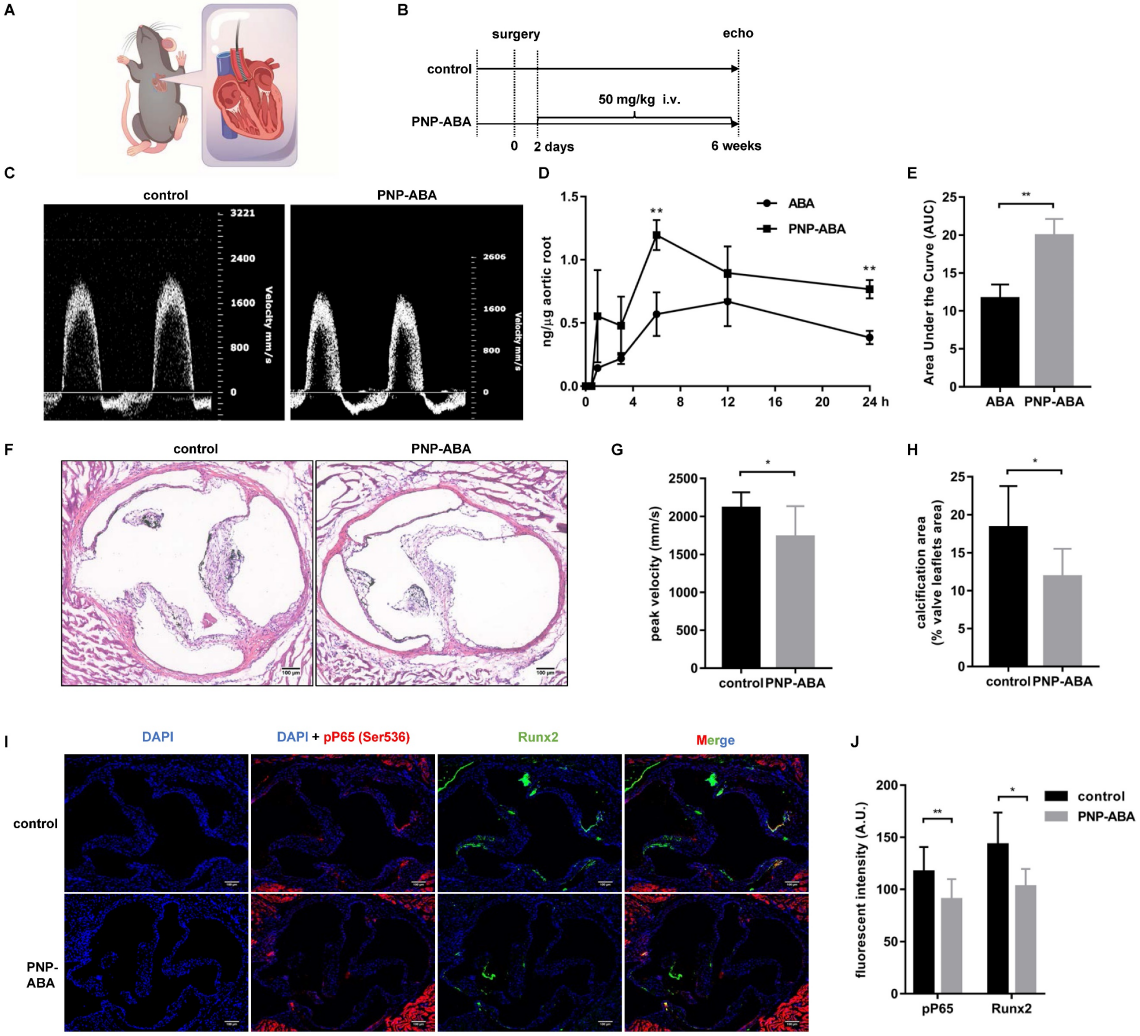
** ABA attenuated calcification in murine wire-induced aortic valve stenosis model.** (**A**) Schematic illustrations of wire-induced aortic valve stenosis model; (**B**) The diagram of the steps of the experiment procedure; (**C**) Results of echocardiogram in control and PNP-ABA injected groups; (**D, E**) After ABA and PNP-ABA injected, the concentration of ABA was detected in mouse aortic root in 24 h (n = 6 per group. Data are presented as the mean ± SEM, **P < 0.01); (**F**) The degrees of calcification were measured by Von Kossa staining (scale bar: 100 µm); (**G**) Quantitative assessment of peak velocity by echocardiography; (**H**) The quantification of Von Kossa results; (**I**) The pP65 and Runx2 was detected by immunofluorescence combined with DAPI staining for nuclei in control and PNP-ABA groups (scale bar: 100 µm). (**G**, **H**, **J**) n = 7 per group. Data are presented as the mean ± SEM, *P<0.05.

A wire-induced aortic valve stenosis model was used to assess the effect of PNP-ABA in vivo (**Figure 6A-B**). The concentration of ABA in the mouse aortic root was higher in the PNP-ABA group than in the ABA group (**Figure 6D-E**). The mouse aortic valve was injured by a wire, and the peak velocity was evaluated by transthoracic echocardiography six weeks postoperatively. Intravenous injection of PNP-ABA (50 mg/kg) significantly reduced transvalvular jet velocity in mice (**Figure 6C, G**). Mouse aortic valves were collected for von Kossa staining. PNP-ABA reduced the calcification area compared to the control group (**Figure 6F, H**), as well as the Runx2 expression level and phosphorylation of P65 in the mouse aortic valve (**Figure 6I-J**). Murine aortic valve can develop to stenosis and calcification 6 weeks post-surgery compared to sham group (**Figure S4B, E**). Echocardiogram assay was performed over time after the establishment of murine wire-induced aortic valve stenosis model (**Figure S4A, C**). PNP-ABA was injected to mice after the model establishment. Interestingly, the peak velocity had no significant difference between injection and control group (**Figure S4D**), nor in the calcified areas in mouse aortic valve leaflets (**Figure S4E-F**).

## Discussion

This study established im-hVICs to perform consistent and stable drug screening for human VICs calcification. ABA has been identified as a potential negative regulator of calcification in human VICs. High-throughput gene expression analysis revealed that ABA is a potent inhibitor of the NF-κB pathway during human VICs osteogenic differentiation. Moreover, ABA supplementation attenuated aortic valve calcification in a wire-induced aortic valve stenosis model.

Human VICs have been discussed in the literature as an important player in heart valve degeneration and are involved in the pathogenesis of CAVD 23, 24. These cells appear critical for homeostasis regulation. However, on the other hand, they are responsible for aortic valve fibrosis and osteogenic degeneration, which are associated with the activation of developmental transcriptional regulatory pathways 25. Anatomically, the aortic valve is composed of three cusps (leaflets): the left coronary cusp (LCC), right coronary cusp (RCC), and non-coronary cusp (NCC), which are attached to the fibrous ring, forming a crown-like structure 26. The aortic valve cells that populate specific leaflets may have different embryonic origins, as LCC and RCC arise from conical (superior and inferior septal) cushions, whereas NCC arises from the posterior intercalated cushion of the outflow tract 27. This may explain the important functional differences in p-hVICs isolated from different cusps 28. To exclude interference from the heterogeneity of p-hVICs, we established im-hVICs for drug screening (**Figure 1A**). Immortalized human VICs exhibited the same phenotype as p-hVICs when cultured in an osteogenic induction medium. Pro-osteogenic biomarkers, including Runx2, BMP2, and OCN, were increased in both cell types, as well as the calcium deposits shown by alizarin red staining (**Figure 1B-C, E**). Immunofluorescence staining indicated that im-hVICs expressed vimentin, which is a marker of human VICs (**Figure 1D**). However, primary human VICs could not grow into a single-cell clone after seeding at a very low density (**Figure 1D**). There was no significant difference in migration ability between the two cell types (**Figure S1A-B**), but immortalized human VICs had enhanced proliferation ability (**Figure S1C-E**). This is because SV40T activates ribosomal genes in host cells, induces DNA synthesis, and modifies the initiation factors of protein synthesis 29. SV40T alters the cell cycle and proliferation by inhibiting the expression of p53 and RB proteins 30. Apoptotic cells in two groups are lower than 1% (**Figure S1F-G**).

This result indicates that both cell lines are suitable for invitro experiments of CAVD. High-throughput RNA sequencing results further confirmed that the growth and development pathways were activated in im-hVICs (**Figure S2**). In addition, ossification pathways were upregulated in both cell types (**Figure S2B**). These results indicate that im-hVICs can be used as a cell line for human VICs calcification research. Im-hVICs are especially suitable for drug screening research due to their high uniformity. It is also possible to generate a single clone gene editing im-hVICs line since im-hVICs can grow into a single-cell clone after seeding at a very low density (**Figure 1D**). However, im-hVICs are unsuitable for studies on proliferation and metabolism.

With these highly consistent im-hVICs, we performed long-term osteogenic induction culture supplemented with 10 different compounds, which are potential anticalcific drugs in our in-house natural products library. ABA (compound **6**), a fusicoccane diterpenoid isolated from fungus *A. brassicicola*, attenuated the calcification of im-hVICs (**Figure 2A-C**). A previous study indicated that ABA is a potent inhibitor of the NF-κB p65 subunit in a LPS-induced inflammatory model 10. The anticalcific effect of ABA in human VICs is highly correlated with the inflammatory pathway because the osteogenic phenotype of human VICs is characterized by the increased expression of Runx2 and BMP2, and enhanced mineralization is caused by inflammatory changes 31. Transcriptome sequencing analysis was used for global gene expression of im-hVICs following various treatments to investigate the mechanisms by which ABA attenuates the OM-induced calcification of human VICs. The KEGG pathway analysis by transcriptome sequencing was highly enriched in the TNF, NF-κB, PI3K-AKT, and TGF-β signaling pathways (**Figure 3A-B**). Strikingly, IKBKG (IKKγ) and IKBKB (IKKβ), which are key activators of the NF-κB pathway 32, were downregulated following treatment with ABA in human VICs osteogenic induction (**Figure 3C**). It has been reported that in hVICs, activation of the NF-κB signaling pathway directly induces osteogenic differentiation 33. Thus, the NF-κB signaling pathway was chosen for subsequent experiments. As we all know, in mammals, there are five members of the transcription factor NF-κB family: RelA (p65), RelB, and c-Rel, and the precursor proteins NF-κB1 (p105) and NF-κB2 (p100), which are processed into p50 and p52, respectively 34. Molecular docking results indicated that ABA interacts with P65 and P52 (**Figure 3D-E**). Thus, P65 and P52 were chosen for subsequent experiments. In further studies, ABA was found to attenuate the phosphorylation of P65 (Ser536), but there was no significant difference in the expression of P52 (**Figure 4A-C**). The translocation of phosphorylation P65 was also reduced by treatment with ABA (**Figure 4F-G**). Interestingly, ABA inhibited osteogenic differentiation of hVICs in a dose-dependent manner at 5 or 10 μM (**Figure 4D-E**), which is also a non-cytotoxic concentration, as evidenced by the IC_50_ of ABA-treated im-hVICs (**Figure 2E**). Our results showed that ABA inhibited the nuclear translocation of P65 was consistent with the results of previous studies. They also confirmed that P65 is recruited to the BMP2 and Runx2 promoter and directs osteogenic programming in VICs 35, 36. However, ABA failed to reduce calcium deposition in already calcified im-hVICs (**Figure S3**). In our animal study, PNP-ABA had no therapeutic efficacy after the establishment of murine wire-induced aortic valve stenosis model (**Figure S4**). Early endothelial dysfunction and consequent stromal inflammatory responses, including leukocyte recruitment, are involved in the initiation of CAVD. As the disease progresses, CAVD lesions develop more acellular regions characterized by lipid accumulation, calcification and mineralization 37. Activation of NF-κB can increase the production of inflammatory cytokines, leading to exacerbation of aortic stenosis in the early stage. Pre-treatment of ABA in the early stage can prevent CAVD progression as it attenuates NF-κB inflammatory pathway. Once CAVD is established, there is no therapeutic effect with the ABA treatment, because the main signaling pathways activated in the later stage are lipid accumulation and calcification, which are not the functional targets of ABA.

Platelet membrane-coated nanoparticles are biomimetic vehicles that can camouflage cells circulating in the blood. The beneficial properties of this biomimetic approach delineate its applicability in treating and circumventing a variety of diseases, such as cancer, immune disorders, heart disease, phototherapy, and diagnostic applications 38, 39. Many studies have confirmed that platelets can naturally be home to atherosclerotic lesions and exhibit good targeting of sclerotic aortic valves 13, 15. In this study, we coated ABA with a platelet membrane to form therapeutic nanoparticles (**Figure 5A-D**). Primary mouse VICs were isolated to test the ability of PNP-ABA to combine (**Figure 5E-F**). PNP-ABA had a better adhesion ability to primary mouse VICs than ABA alone (**Figure 5G**). The in vivo pharmacokinetic assay also showed that the PNP-ABA group had a higher concentration of ABA in mouse aortic root tissue at 6 and 24 h post-injection (**Figure 6D-E**).

In numerous animal models of CAVD research, the mouse model is the most attractive model for research due to its ease of handling, the ability to have large animal groups, and the availability of various transgenic and knockout strains 40. The ApoE-/-mouse model is the most popular model for CAVD research. However, it commonly takes ≥20 weeks for these models to develop calcification of valve leaflets, and not all animals develop hemodynamically significant thickening 37. In 2014, Honda et al. used a spring wire as a new model of aortic valve calcification. Mice that have undergone surgery can develop aortic stenosis within one week 22. In 2019, Niepmann et al. modified and expanded upon the original protocols and developed distinct models with mild, moderate, and severe wire injuries 41. Murine wire-induced aortic valve stenosis is an ideal model for investigating the role of inflammation during aortic valve calcification. In our study, PNP-ABA treatment reduced the transvalvular peak jet velocity of surgery mice, as well as calcium deposition, as shown by von Kossa staining (**Figure 6C, F-H**). Runx2 expression level and phosphorylation of P65 were also alleviated in the mouse aortic valve area following treatment with PNP-ABA (**Figure 6I-J**). These results were consistent with the previous studies which confirmed that down-regulation of NF-κB pathway attenuates CAVD progression 34. Together, our results indicate that ABA could be a potential therapeutic compound to prevent aortic valve calcification.

## Conclusions

The establishment of im-hVICs is not only beneficial to the study of CAVD, but also provides a convenient method for us to prepare the immortal cell line. With this cell line, we can do more large-scale drug screenings related to human aortic valve disease. It is also worth noting that platelet membrane-coated nanoparticles specifically targeting the aortic valve may be a better drug delivery modality. Finally, the mouse wire-induced aortic stenosis model provides a promising animal model for studying inflammation in CAVD. Most importantly, our study highlights ABA as a promising candidate for preventing the progression of CAVD.

## Supplementary Material

Supplementary figures.

## Abbreviations

ABA: alterbrassicene A
VIC: valve interstitial cell
CAVD: calcific aortic valve disease
p-hVICs: primary human valve interstitial cells
im-hVICs: immortal human valve interstitial cells
LCC: left coronary cusp
NCC: non-coronary cusp
RCC: right coronary cusp
mVICs: mouse valve interstitial cells
PNP-ABA: alterbrassicene A-loaded platelet membrane-coated nanoparticles
OM: osteogenic induction medium
