# Supplementary material for: Platelet membrane-coated alterbrassicene A nanoparticle inhibits calcification of the aortic valve by suppressing phosphorylation P65 NF-κB
Source: Theranostics. 2023 Jun 26;13(11):3781–93. doi: 10.7150/thno.85323 (PMC10334836; doi:10.7150/thno.85323)
Supplement: Supplementary file 1 — Supplementary figures. [file thnov13p3781s1.pdf]

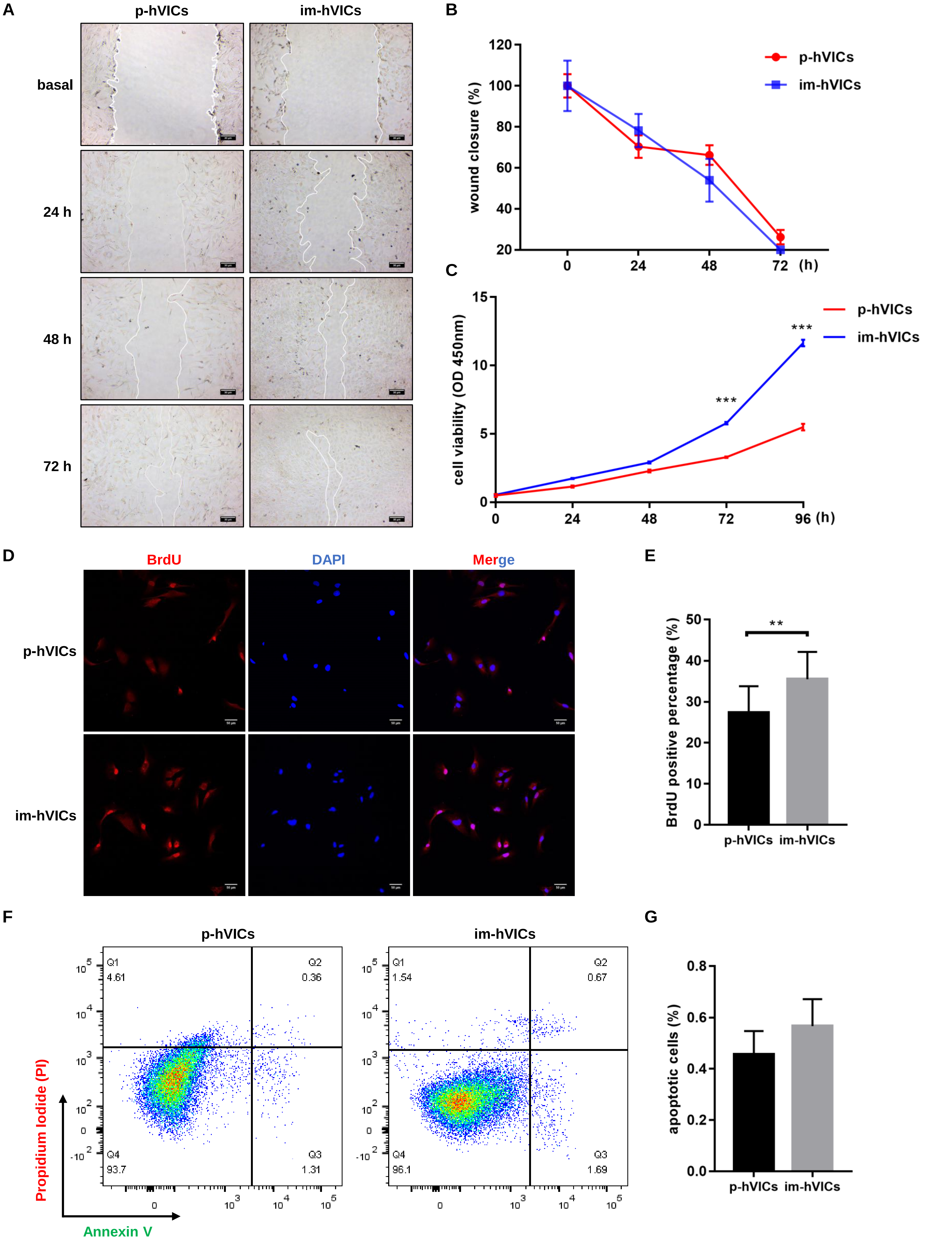

Figure S1. Performance comparison between p-hVICs and im-hVICs.

(A, B) Scratch assay and quantification on p-hVICs and im-hVICs cells (scale bar: 30 μm); (C) The cell viability was determined. (n = 6 per group. Data are presented as the mean ± SEM, ***$P < 0.001$); (D, E) BrdU staining and quantification of p-hVICs and im-hVICs (n = 3 per group. Data are presented as the mean ± SEM, **$P < 0.01$, scale bar: 50 μm); (F, G) Apoptosis assay and quantification of p-hVICs and im-hVICs (n = 3 per group. Data are presented as the mean ± SEM).

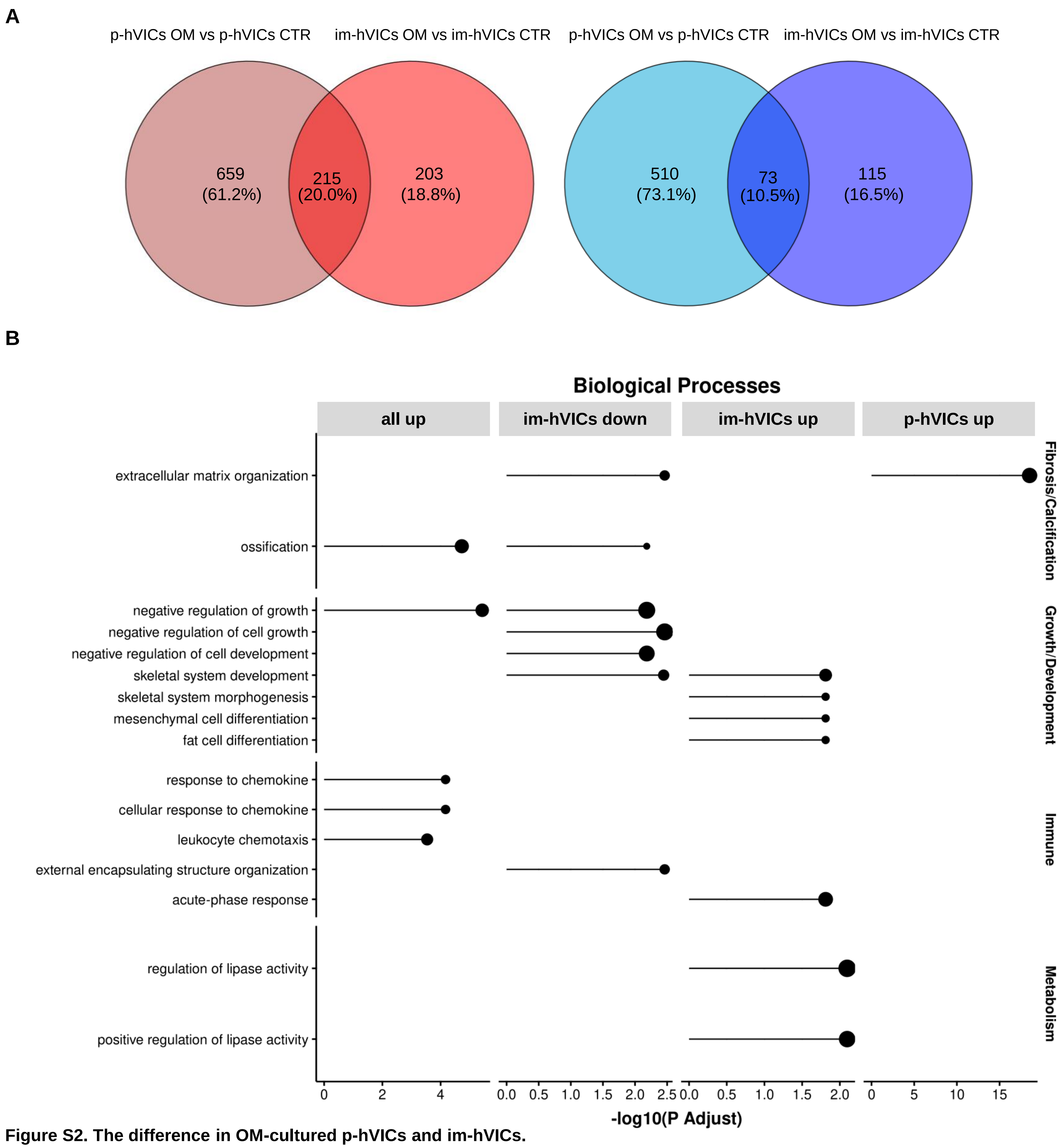

Figure S2. The difference in OM-cultured p-hVICs and im-hVICs.
(A) The common regulated genes in OM-cultured p-hVICs and im-hVICs; (B) Biological processes enriched in OM-cultured p-hVICs and im-hVICs.

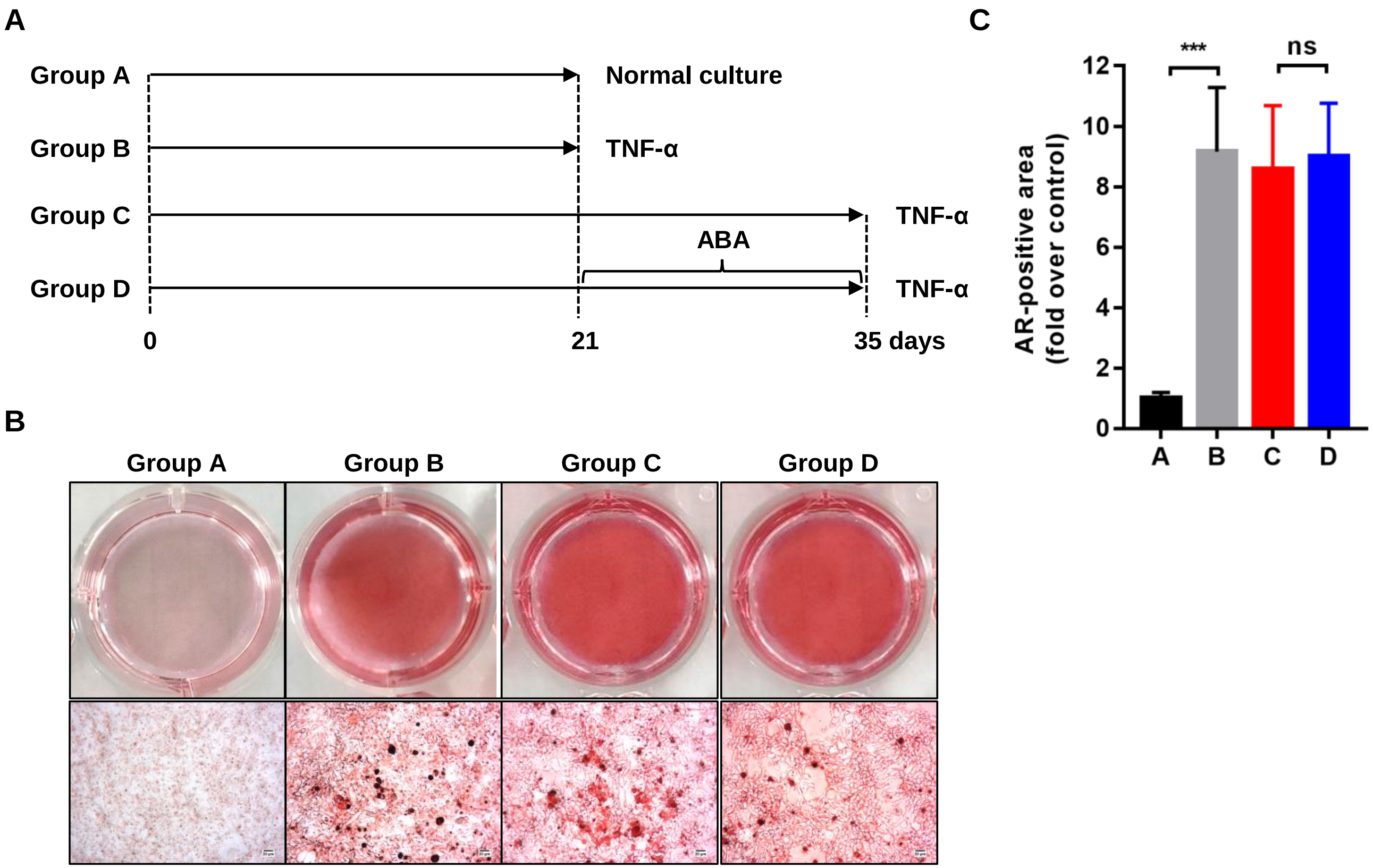

Figure S3. ABA does not reverse calcium deposition in already calcified im-hVIC. (A) The diagram of the steps of the experiment procedure; (B) The calcium deposition of four groups was detected by alizarin red (scale bar: 20 μm); (C) The alizarin red positive area were quantified in im-hVICs (n = 6 per group. Data are presented as the mean ± SEM, $^{***}P < 0.001$, ns indicates no significant).

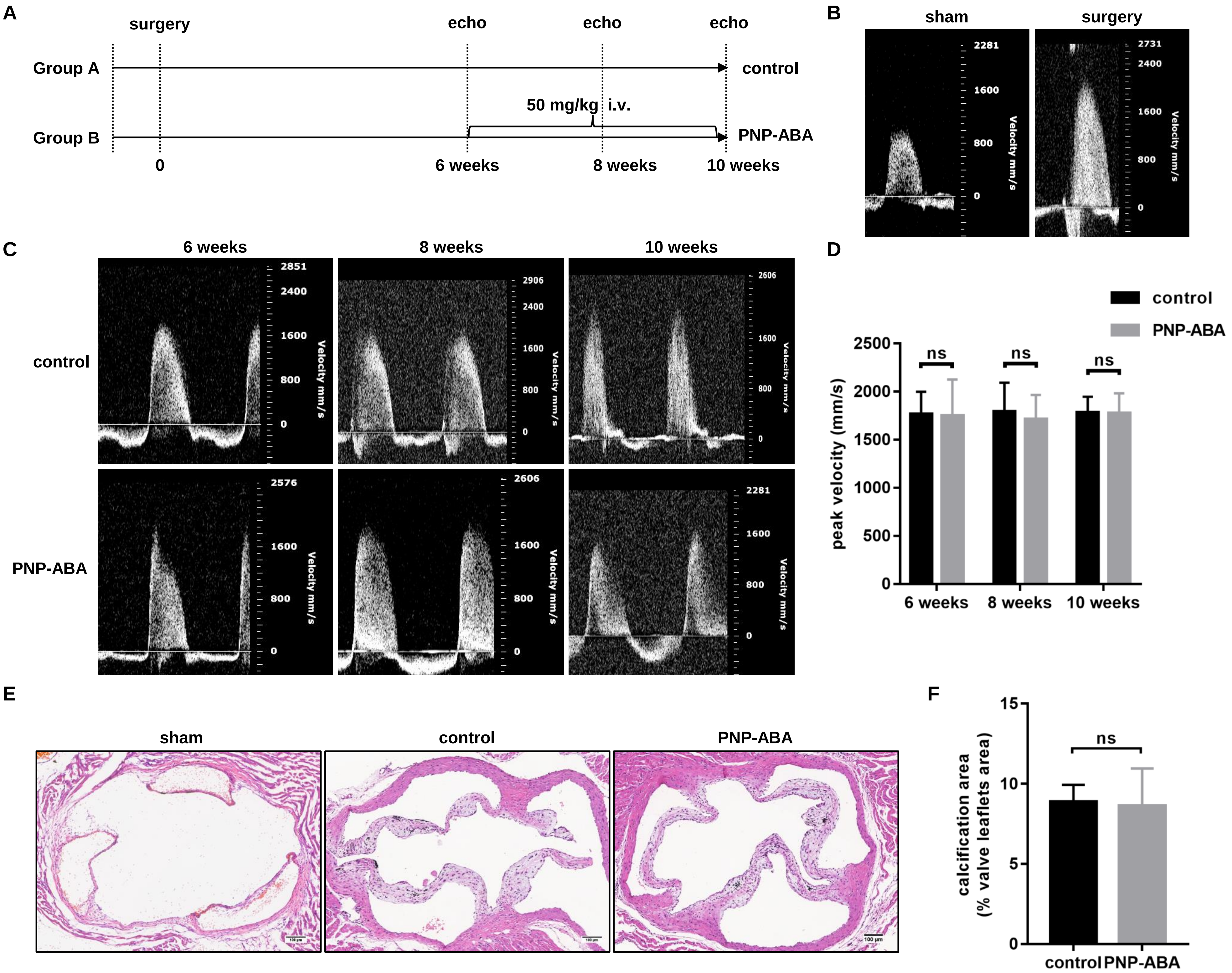

Figure S4. PNP-ABA had no therapeutic efficacy after the establishment of murine wire-induced aortic valve stenosis model.
(**A**) The diagram of the steps of the experiment procedure; (**B**) Results of echocardiogram in sham and surgery group 6 weeks post surgery; (**C**) Results of echocardiogram in control and PNP-ABA injected groups over time; (**D**) Quantitative assessment of peak velocity by echocardiography over time (n = 5 per group. Data are presented as the mean ± SEM, ns indicates no significant); (**E**) The degrees of calcification were measured by von Kossa staining (scale bar: 100 μm); (**F**) The quantification of von Kossa results (ns indicates no significant).
